# Indirect protein quantification of drug-transforming enzymes using peptide group-specific immunoaffinity enrichment and mass spectrometry

**DOI:** 10.1038/srep08759

**Published:** 2015-03-04

**Authors:** Frederik Weiß, Anke Schnabel, Hannes Planatscher, Bart H. J. van den Berg, Bettina Serschnitzki, Andreas K. Nuessler, Wolfgang E. Thasler, Thomas S. Weiss, Matthias Reuss, Dieter Stoll, Markus F. Templin, Thomas O. Joos, Katrin Marcus, Oliver Poetz

**Affiliations:** 1 NMI Natural and Medical Sciences Institute at the University of Tübingen, Markwiesenstr. 55, Reutlingen, Germany; 2 Medizinisches Proteom-Center, Ruhr-University Bochum, Bochum, Germany; 3 Department of Traumatology, Eberhard Karls Universität Tübingen, Tuebingen, Germany; 4 Department of Surgery, Ludwig-Maximilians-University, Munich, Germany; 5 Department of Pediatrics and Juvenile Medicine, Regensburg University Hospital, Regensburg, Germany; 6 Center Systems Biology, University of Stuttgart, Stuttgart, Germany; 7 University of Applied Sciences, Albstadt Sigmaringen, Germany

## Abstract

Immunoaffinity enrichment of proteotypic peptides, coupled with selected reaction monitoring, enables indirect protein quantification. However the lack of suitable antibodies limits its widespread application. We developed a method in which multi-specific antibodies are used to enrich groups of peptides, thus facilitating multiplexed quantitative protein assays. We tested this strategy in a pharmacokinetic experiment by targeting a group of homologous drug transforming proteins in human hepatocytes. Our results indicate the generic applicability of this method to any biological system.

Selected reaction monitoring (SRM) is a valuable tool in targeted proteomic approaches^1^. Proteins in biological samples are enzymatically fragmented down to peptides. Then peptides are chromatographically separated and analyzed by a triple quadrupole (QQQ) mass spectrometer. Here selected peptide precursor ions of target proteins are selectively isolated. The precursor ions are subsequently fragmented in a collision cell. Proteins are then indirectly identified and monitored by a second filter process for peptide-specific secondary ions (transitions). Indirect protein quantification is achieved by simultaneously measuring a defined, spiked amount of corresponding isotopically-labeled (C13/N15) reference peptide^2^. The sample consumption is low and a high number of proteins can be analyzed in parallel^3^. This type of mass spectrometric measurement is highly precise. However, the release of the selected proteotypic peptide during proteolysis can vary and has to be investigated target by target. Therefore, the quantification derived from the reference peptide is not absolute; however, quantification is in fact absolute when complete tryptic fragmentation without a single missed cleavage site is achieved. The use of metabolically labelled recombinant proteins will improve this in future, since a protein standard undergoes all sample processing steps^4^. However, the sensitivity of SRM-based protein assays is limited: this is caused e.g. by ion suppression effects that arise from the sample amount and complexity^5^. Peptide-specific antibody sets are used in a parallelized immunoprecipitation step to reduce sample complexity and thereby concurrent suppression effects. As a consequence sensitivity and throughput is greatly increased^6^,^7^. Although this method does not match the speed and sample throughput of other antibody-based methods, such as sandwich immunoassays^8^, its specificity is excellent. This highly specific protein detection is based on two independent lines of evidence: immunoaffinity capture and MS/MS readout. However, broad application of this immunoaffinity MS strategy in proteomic experiments has been restrained by the lack of suitable antibodies. The shortage of specific antibodies is still a limiting factor although rapid progress is being made in large-scale projects targeting the generation of antibodies on a proteomic-wide scale^9^,^10^.

Recently we demonstrated that antibodies with a short C-terminal peptide epitope (triple X proteomics antibody) can be applied to enrich peptides that have a common C-terminal motif^11^. This strategy substantially reduces the number of antibodies required for a given set of proteins^12^. In the present study we combined this type of antibody with SRM to create a quantitative assay set-up for profiling key molecules of the hepatic drug metabolism in human which are relevant to preclinical research and drug-drug interaction profiling: the subfamily CYP3A of the human cytochrome P450 enzymes (CYP) and the ATP-binding cassette (ABC) transporter multidrug-resistance protein 1 (MDR1/P-glycoprotein). Several publications have described SRM-based assays for the quantification of the cytochrome P450 enzymes (CYP) in liver, hepatocytes and hepatoma cell lines^13^,^14^,^15^,^16^,^17^. The approaches described involve laborious, time-consuming steps during the preparation of microsomes for enrichment of the low expressed CYP isoforms as required for a reliable quantification. Moreover, CYPs or other low abundant proteins can be enriched by their molecular weight, using SDS-polyacrylamide electrophoresis and quantified by LC-SRM-MS(/MS) analysis^17^,^18^. We have established a method for quantifying three homologous CYP enzymes (CYP3A4/43, CYP3A5, CYP3A7) and the drug transporter multidrug-resistance protein 1 (MDR1) directly from a low amounts of human hepatocyte lysates: only one TXP-antibody is required in the immunoaffinity enrichment step prior to MS-based quantification.

## Results

**Figure 1:**
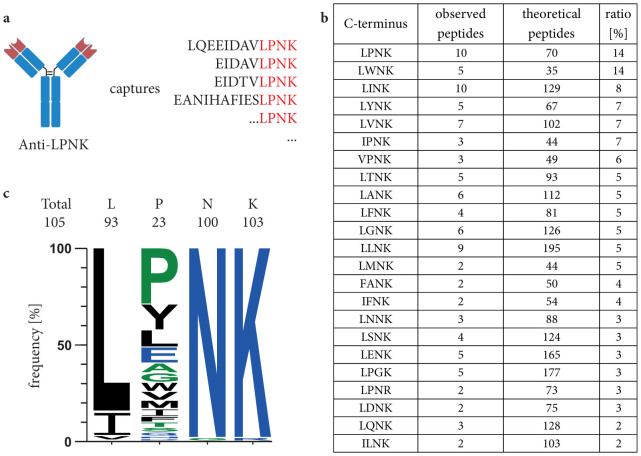
(a) Peptide group-specific antibodies. TXP antibodies target peptide subsets of global tryptic digests that share the same terminus. (b) Frequency of C-termini in a peptide pool immunoprecipitated by the antibody anti-LPNK. Peptides were immunoprecipitated from 50 μg tryptically digested hepatocyte and analyzed by high resolution tandem-mass spectrometry. Theoretical number of peptides harboring the respective C-termini were calculated from an *in silico* digest of the Uniprot database (UniProt Homo Sapiens Reference Proteome Set database (2014 Release 1)). (c) Amino acid frequency in the binding motif of the antibody, calculated based on the high resolution tandem-mass spectrometry analysis.

First, we performed a C-terminal-anchored alignment of all peptide sequences derived from an *in-silico* tryptic digest of the CYP3A sub-family relevant for main drug transforming processes^19^. We identified three peptides that were suitable for SRM analysis: LQEEIDAVLPNK, EIDAVLPNK and EIDTVLPNK. Each of them carry the C-terminus LPNK. Further bioinformatic analysis revealed that a proteotypic fragment of the drug transporter MDR1 also contains this sequence and can be captured by an anti-LPNK-antibody. This multi-specific antibody (Figure 1a) was generated and purified as described previously^11^. We employed the synthetic peptide of each target to identify the optimal transitions to be monitored (see Supplemental Table 1 and 2 for transitions of the respective peptides). Three transitions, per peptide were defined for unequivocal peptide detection and the most intensive was selected to be the ‘quantifier’ transition. It should be noted, that the peptide LQEEIDAVLPNK is also present in the sequence of the enzyme CYP3A43. However, no or only very low expression in hepatocytes respectively liver was observed previously^14^,^20^.

**Figure 2:**
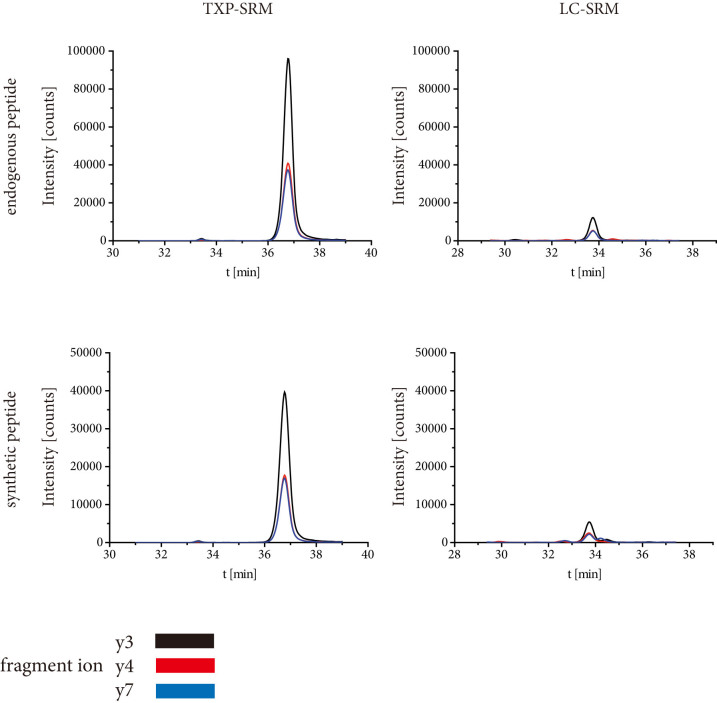
Comparison of signals of endogenous cytochrome P450 3A4/43, (b) 3A5, (c) 3A7, and (d) MDR-1 peptide and synthetic standard. Ten μg hepatocyte protein extract was digested and spiked with 100 fmol synthetic standard. The samples were either analyzed by TXP-SRM or LC-SRM workflow. Ion chromatograms of the peptide fragments y3, y4 and y7 are shown.

To analyze the performance and the impact of multi-specific immunoprecipitation, we compared the results generated by means of a standard LC-SRM method with the TXP-LC-SRM method which uses the antibody specific for LPNK. We thus digested 20 μg protein extract from human hepatocytes using trypsin as the fragmenting reagent. The processed sample was divided and analyzed side by side with the two different workflows. Only the more abundant enzyme, CYP3A4/43 and CYP3A5 could be unambiguously identified and quantified through the LC-SRM-based method without any enrichment step. Either no transition or only one was observed in the case of the CYP3A7- and MDR-1-specific peptides: thus, they could not be quantified. With the TXP-SRM approach, all three transitions were detectable and all four proteins were quantified (Supplemental Table 3, Figure 2, Supplemental Figure 1A–C) from the hepatocyte extract. This increase in sensitivity can be explained by the dramatic decrease of peptide complexity, which reduces ion suppression effects.

**Figure 3:**
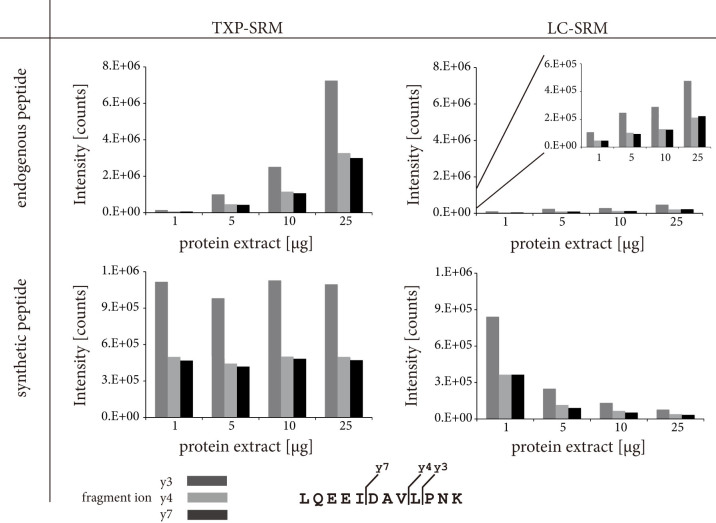
Comparison of integrated signals of endogenous cytochrome P450 3A4 peptide and synthetic standard. One, five, ten or 25 μg hepatocyte protein extract was digested and spiked with 100 fmol synthetic standard. The samples were either analyzed by TXP-SRM or LC-SRM workflow. Integrated signals of the peptide fragments y3, y4 and y7 are plotted against the analyzed amount of protein extract.

Matrix effects on the two different workflows were studied using four different amounts of hepatocyte extract (1, 5, 10 and 25 μg total protein). In the TXP-SRM workflow, the signal intensity of the reference peptide was not affected by the amount of initial sample load. This demonstrates that the quality of signal and quantification is greatly improved by introducing a rapid immunopreciptitation step before LC-SRM analysis (Figure 3, Supplemental Figure 2). In contrast, in the LC-SRM workflow the increasing amounts of tryptically-fragmented hepatocyte sample resulted in decreasing signal intensity of the internal reference; whereas, the signal intensity of the endogenous peptide increased (Figure 3). This shows that the relation between the sample quantity (endogenous peptide) and the peptide standard needs to be carefully adjusted before routine quantification exclusively using LC-SRM is done. In the TXP-SRM approach it is not necessary to balance sample amount and internal standard because no signal suppression was observed. Moreover, we compared transition ion intensity ratios for each peptide since this is an additional mean for the evaluation of the specificity in peptide quantification by single reaction monitoring^5^. These ratios resulting from the endogenous and the synthetic peptide should not differ from each other. However, in some cases components of the sample influence these ratios by co-eluting molecules generating interferences in one of the monitored transitions. We observed no such interferences for any of the immunoprecipitated peptides (Supplemental Table 4). However, in the LC-SRM approach only the CYP3A4 peptide surrogate achieved acceptable values (Supplemental Table 3).

Since most antibody-based immunoassay approaches target only one molecule, our aim was to demonstrate how an antibody can capture four different analyte peptides with equal efficiency. Therefore, we mixed 1 pmol of each standard peptide (non-labelled version) with 1 μg antibody and analyzed the captured fraction by eluting the peptides into the same amount of standard peptide (isotopically-labelled version). We observed for each peptide an amount of 400 fmol of the initial peptide quantity in the eluate by forming the signal ratio of labeled-to non-labeled peptide. In sum for all four peptides the antibody's capacity was 1.6 pmol LPNK-peptide per μg antibody. The theoretical number of binding sites for one μg antibody is 13 pmol and if this number is used, the antibody's capacity is 12%. More importantly, we found no evidence of preference towards one of the four peptides, which demonstrates that peptide group-specific enrichment of analytes with a shared epitope is feasible. (Supplemental Figure 3).

We also determined the technical intra- and inter-day variation. A 10 μg hepatocyte protein extract from one hepatocyte donor was processed and analyzed three times on the same day for variance calculation. We calculated the inter-day variation of three independent experiments carried out on three different days. Depending on the analyte, intra-day variation ranged from 6 to 11% and inter-day variation from 9 to 19% (detailed values are given in table 1).

The lower limit of quantification (LLOQ) was determined in an analyte-free commercially available buffer preparation containing proteolytically fragmented gelatine. The LLOQ was determined according to the FDA's recommendation for bioanalytical method validation^21^. Thus, the mean value for accuracy (variation) and precision (recovery) should be within 15%. An LLOQ is reached when values exceed 20%. Starting from 100 nM, isotopically-labeled peptides were serially diluted eight times at a ratio of 1:3 in digested fish gelatine, while sequence-identical endogenous peptides were kept constant at a concentration of 10 nM. Experiments were performed in triplicate. After precipitation by the anti-LPNK antibody, the peptides were measured by targeted selected ion monitoring (tSIM), using high-resolution tandem mass spectrometry (LC-MS/MS, QExactive). While the LLOQ for the MDR1 peptide was reached at 140 pM or 1.4 fmol absolute peptide amount, the limits for the CYP3A4/A43, Cyp3A5 and CYP3A7 were three times lower and 46 pM or 460 amol peptide (Supplemental Figure 4).

To investigate the specificity of the antibody (on- and off-target binding), we analyzed an immunoprecipitated peptide pool, starting with 50 μg hepatocyte protein extract using non-targeted liquid chromatography high resolution tandem mass spectrometry (LC-MS/MS, Q Exactive). In addition to two analytes, CYP 3A4 and MDR-1 described above, the experiment revealed 8 peptides containing the targeted LPNK motif at the C-terminus (Figure 1b and c, Supplemental Table 5). The surrogate peptides for CYP3A5 and 3A7 were not detected in these analyses. We also identified 89 additional peptides with different motifs for one amino acid and six peptides differing in two amino acids. This mainly affected leucine and proline. Our findings provide evidence for a strong preference for LPNK-motif binding (Figure 1b).

**Figure 4:**
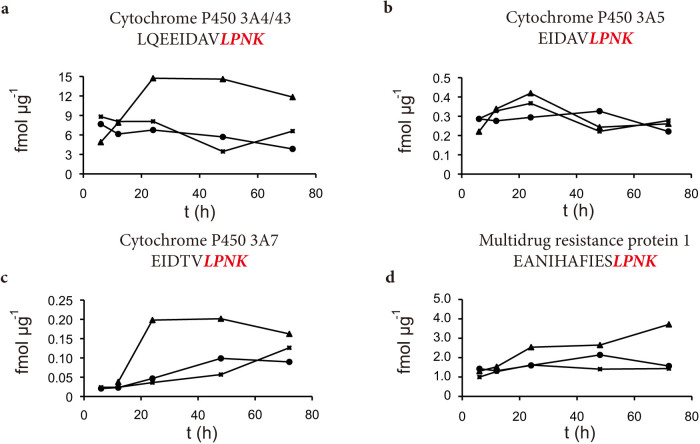
Induction of cytochrome P450 3A4, 3A5, 3A7, and multi-drug resistance protein 1. Human hepatoycte cultures (6-well format, 3 days after isolation) were treated with 60 μM atorvastatin (triangle), 30 μM pravastatin (circle) and solvent control (cross, DMSO) for 6, 12, 24, 48, and 72 hours. Cells were harvested and homogenized. 10 μg protein extract of each time point was analyzed using the TXP-SRM assays for cytochrome P450 (a) 3A4, (b) 3A5, (c) 3A7, and (d) multi-drug resistance protein 1.

We applied this multiplex assay in a pharmacokinetic experiment. Primary hepatocytes were treated over a three-day period with two different statins, pravastatin and atorvastatin. Cells were harvested after 6, 12, 24, 48, and 72 hours and analysed with the established TXP-SRM assay (Figure 4). Atorvastatin is known to induce CYP3A4 via the nuclear pregnane X receptor (PXR)^22^. CYP3A4 expression is not influenced by pravastatin treatment, as Feidt et al. described^22^. The impact of atorvastatin and pravastatin on the protein expression of CYP3A5, CYP3A7 and MDR-1 has not been described so far. In our experiment we observed no induction of the monitored enzymes and the transporter in the pravastatin-treated cells, as compared to the solvent control (DMSO) (Figure 4a–d). We observed three-fold and ten-fold induction of CYP3A4 (Figure 4a) and 3A7 (Figure 4c) by atorvastatin, with a peak after 24 h however, CYP3A5 was not affected (Figure 4b). A late two- to three-fold induction by atorvastatin was observed in MDR-1 protein expression over time (Figure 4d). Our results confirm prior findings relating to mRNA expression and enzymatic activity analyses for CYP3A4 induction by atorvastatin. To our knowledge, we are the first to report an increase in CYP3A7 and MDR-1 protein levels after atorvastatin treatment (Figure 4c and d). The induction mechanism most likely involves the PXR mediated transcriptional regulation because functional PXR binding sites in the 5′–flanking region of both genes were identified previously^23^,^24^.

## Discussion

We established a new multiplex MS-based immunoassay (TXP-SRM) using one antibody. The antibody's specificity towards the short C-terminal peptide sequence LPNK allows the quantification of three drug metabolizing enzymes and one drug transporter from a minute amount of sample. Through an analysis of statin-treated human hepatocytes, we indicated the relevance of this method to pharmacokinetic investigations. By introducing a rapid immunoaffinity step into the LC-SRM-workflow sensitivity was improved more than ten-fold and a direct analysis of the transporter and the three drug-metabolizing enzymes without membrane fractionation or microsome preparation facilitated. In typical induction experiments the cytochrome isoforms 1A2, 2B6, 2C8, 2C9, 2C19, and 2E1 are investigated in addition to CYP 3A4 by mRNA expression analyses and/or activity assays^25^. Future immunizations targeting the termini FSGR (2B6, 2C8, and 2E1), LAER (2C9 and 2C19) and VIGR (1A2) will allow assay development for those enzymes. These assays might allow the monitoring of induction kinetics of a larger cytochrome P450 set on protein level, because problems caused by enzyme inhibition or mRNA degradation are avoided.

Apart from the analysis of homologue protein families, the strength of this approach lies in its generic applicability to any biological system as it offers the advantage of using a small number of antibodies for the quantification of a large number of proteins.

## Methods

### Isolation and cultivation of primary human hepatocytes

Primary hepatocytes were isolated and cultured as described previously^26^. Preparations from human liver resections from patients undergoing partial hepatectomy were performed according to the guidelines of the charitable state-controlled foundation HTCR (Human Tissue and Cell Research) Regensburg, Germany and the institutional guidelines for liver resections of tumor patients with primary or secondary liver tumors, Technical University Munich, MRI, Munich, Germany. The scientific use of human hepatocytes was approved by the local ethics committees of the Ludwig-Maximilians-University of Munich^27^ and the Technical University Munich, MRI, Munich^28^, Germany. Written informed consent was obtained from all patients.

Hepatocytes were plated at a density of 1.5 * 10^6^ cells/well on collagen-coated 6-well plates. Cells were allowed to settle down to the collagen layer. Culture media was replaced by serum-free Williams Medium E (Pan-Biotech-GmbH), supplemented with albumin (0.1% (v/v)), after transport. Before the induction experiment was started, cells were starved for 24 h at 37°C in a humidified chamber with 95%/5% air/CO_2_, penicillin/streptomycin (100 U/mL), stabilized L-glutamine (2 mM), dexamethasone dihydrogenphosphate (0.025% (v/v)) and ITS-X (5 mg insulin, 3.35 μg sodium-selenite, 2.75 mg transferrin and 1 mg ethanolamine)(Invitrogen), further referred to as SFM.

Cells were treated with 60 μM atorvastatin 30 μM pravastatin and solvent control (DMSO) in SFM for 6, 12, 24, 48, and 72 hours. Cells were harvested in PBS, supplemented with protease inhibitors (Roche, Complete Mini EDTA-free). Cell suspensions were spun down for 5 min at 500 rcf at 4°C and washed once in 150 μL NaPP-buffer (0.1 M, pH 7.4), containing 250 mM sucrose and protease inhibitors (Complete Mini EDTA-free, Roche). Cells were homogenized by ultra-sonification and lyophilized. Total protein content was determined by amino acid analysis on an HPLC system (2695, Waters) using the 6-aminoquinolyl-N-hydroxysccinimidyl carbamate (AQC) reagent and calculated as described previously^29^,^30^.

### Peptide Standards

Peptide standards (Thermo Fisher) were quantified by amino acid analysis on an HPLC system (2695, Waters) using the AccQ Tag derivatisation protocol (Waters) as described previously^29^,^30^.

### Protein Digestion

Proteins were precipitated by adding acetone to 80% v/v and were incubated overnight at −80°C. Samples were centrifuged at 10.000 rcf at 4°C for 5 min. Supernatant was discarded and proteins were air-dried for 5 min. An amount of 100 fmol per internal standard peptide was added after resolving the proteins in 8 M urea, 0.05% 3-((1-(Furan-2-yl) undecyloxy) carbonylamino) propane-1-sulfonate (ProteaseMAX, Promega) in 50 mM ammonium bicarbonate pH 7.4. Proteins were reduced with 5 mM dithiothreitol (DTT) in the presence of 0.05% ProteaseMAX (Promega) for 20 min at 56°C and alkylated with 15 mM iodacetamide (IAA) in 50 mM ammonium bicarbonate for 15 min at RT in the dark. Finally samples were digested with trypsin using an enzyme:substrate ratio of 1:10 at 42°C for 4 h on a shaker. Enzymatic reaction was terminated by boiling for 5 min.

### Immunoprecipitation

Enzymatically fragmented proteins were mixed with 2 μg anti-LPNK antibody and incubated at 500 rpm at 30°C for 1 h on a shaker (Eppendorf). Peptide-antibody complexes were precipitated by adding 10 μL protein G-coated magnetic microspheres (Invitrogen). Beads were washed using disposable 200 μL pipette tips and a ring magnet. Five wash cycles were performed with 100 μL PBS, supplemented with 0.3% n-octyl-β-glucoside (SIGMA Aldrich) followed by a final wash using PBS only. Peptides were eluted by applying 20 μL 1% formic acid.

### Peptide desalting

C18 material containing tips (OMIX, Agilent) were pre-conditioned 5 times with 100 μL 50% acetonitrile and 5 times with 100 μL 0.1% trifluoric acid (TFA). Peptides were extracted from the sample by pipetting 100 μL 8 times. Peptide elution was carried out in two steps using 15 μL 55% acetonitrile, 0.1% TFA. The peptide solutions were dried in a vacuum centrifuge and re-solubilized in 25 μL 0.1% TFA, 1% acetonitrile.

### LC-SRM

Peptide quantification was performed on a hybrid triple quadrupole ion trap mass spectrometer (4000QTrap; ABISciex) equipped with a nanoelectrospray ion source. Prior MS-analysis desalted or immunoprecipitated peptide samples were separated on a nanoliter flow HPLC system (Ultimate, Dionex) using two precolumn as described previously^31^. After injection (15 μL), peptides were trapped and desalted on a pre-column (0.3 mm I.D. × 5 mm PepMap™, Dionex) at a flow rate of 30 μL/min in 0.1% TFA for 6 min. Peptides were transferred to the separation column (75 μm I.D. × 250 mm PepMap™ column, Dionex) and separated by applying a linear gradient of mobile phase (A: 0.1% formic acid, B: 84% acetonitrile/0.1% formic acid) from 5% B to 40% B over a period of 35 min with a flow of 300 nL/min.

The mass spectrometer was run in multiple-reaction monitoring mode. Peptide-specific tuned declustering potential (DP), entrance potential (EP), collision cell energy (CE), collision cell exit potential (CXP), and retention times are given in supplemental table 2. Three transitions per surrogate peptide were monitored. The scheduled option was employed for all data acquisition with a target scan time of 3 s, cycle time of 30 min and an 8 min detection window. Data was processed using MultiQuant TM 3.0 (ABSciex) applying the MQL algorithm for peak integration. Retention time window was set to 4 min and a three-point smooth with a peak splitting factor of 2 applied. No noise subtraction was done. The endogenous amounts of CYP3A4, CYP 3A5, CYP3A7 and MDR1 were calculated from integrated areas of the respective ion-chromatogram of the surrogate peptide transitions. Briefly, the average of the three peak area ratios were formed: internal standard peptide transitions: endogenous peptide transitions.

### Epitope analysis by LC-MS

To investigate the specificity of the anti-LPNK antibody LC-Full-MS analyses of three immunoprecipitation replicates from digested hepatocyte lysates were performed. We incubated 5 μg anti-LPNK antibody with 50 μg tryptically digested hepatocyte lysate. Peptide antibody complexes were precipitated with 25 μL protein G-coated magnetic microspheres (Invitrogen). Peptides were eluted in 20 μL 1% formic acid. The entire eluate (20 μL) was injected and separated on a nanoliter flow HPLC system (U3000, Dionex). Peptides were loaded on an Acclaim PepMap100 C18 μ-pre-column (0.3 mm I.D. × 5 mm, 5 μm particles, Dionex) for 3 min at a flow rate of 20 μL/min in 0.1% TFA. The peptides were separated for 20 min by an Acclaim PepMap RSLC C18 (75 μm I.D. × 150 mm, 2 μm particles, Dionex) using a linear gradient from 5% to 55% B at a flow rate of 300 nl/min at 40°C. A Full-MS analysis was performed on a QExactive™ Plus Orbitrap Mass Spectrometer (Themo Scientific). In MS mode the resolution was set to 70.000, the scan range from 300–2000 *m/z*, the AGC-target limited to 3e6, and the injection time to 100 min. For MS2 mode a top 10 method was selected, the precursor AGC-target limited to 5e5, the maximum injection time set to 60 min, and the normalized collision energy adjusted to a value of 25%. No underfill ratio was chosen and only 2+, 3+, and 4+ charged ions included. The dynamic exclusion time was set to 5.0 s. For identification both Mascot- and SEQUEST-algorithm were chosen for searching in the UniProt Homo Sapiens Complete Proteome Set database (28 august 2013). Two missed cleavage sites were tolerated. The precursor mass tolerance was set to 5 ppm, the fragment mass tolerance to 0.05 Da. Carbamidomethylation of cysteins were allowed as static modification, methionine oxidation as dynamic modification.

For modeling the WebLogo (WebLogo 3, http://weblogo.threeplusone.com/create.cgi), all peptide identifications of the digest-immunoprecipitation triplicate were combined. Unspecific peptide binding to antibody and protein G was determined by analyses of immunoprecipitation experiments using (i) protein G bead and sample and (ii) protein G-beads and antibody without sample (measured in triplicates). Those peptides were considered as background and removed from the identification list. Peptide duplicates were removed. Identifications with at least two identical c-terminal four-amino acid tags were further considered for modelling the epitope specificity. The weighting of amino acid presence was related to the total number of peptide spectrum matches (PSM, ProteomeDiscoverer 1.3, Thermo Scientific) across the triplicate. Parameters of the WebLogo software were kept default except for “units”, which was set to “probability”.

### Dilution series in buffer

Isotopically-labeled peptides were diluted in digested fish gelatin, 50 mM Tris-HCI, 150 mM NaCl, and pH 7.4 (Blocking Reagent, Roche) while sequence-identical endogenous peptides were kept constant at a concentration of 10 nM. Starting from a concentration of 100 nM, the isotopically-labeled peptides were serially diluted eight times at a ratio of 1:3. According to the precipitation procedure, 10 μL of each dilution step was incubated with 1 μg anti-LPNK. For the [recommended] peptide sequence, an absolute peptide amount ranging from 1000 fmol to 0.15 fmol was provided for immunoprecipitation.

### Dilution series in complex matrix

Isotopically-labeled peptides were diluted in hepatocyte extract. In an immunoprecipitation procedure, 1 μg anti-LPNK was incubated with 10 μg digested hepatocyte extract plus an isotopically-labeled peptide amount ranging from 1000 fmol to 0.15 fmol.

### Peptide quantification using LC-coupled targeted Single Ion Monitoring (LC-tSIM)

For quantification, the identical LC-setup was used as described in “Epitope analysis by LC-MS”. Instead of a linear gradient, a step gradient starting at 15% B and ending after 8 min at 40% B was chosen. On the QExactive Plus Orbitrap Mass Spectrometer, a tSIM method was applied to quantify peptides at the parent ion level. The resolution was set to 70,000, the scan range was between 300 and 2000 m/z, and the AGC target was set at a value of 5e6. The injection time was adjusted to 120 min. Loop count and MSX count were set to 2 respectively. An inclusion list registering the masses of endogenous and isotopically-labeled peptides was defined. For ensuring a high number of data points, the recording time for each signal pair was limited to 0.3 min prior and following the expected elution time. Data-dependent MS/MS was performed using a resolution of 17,500, an AGC-target of 2e5, and a maximum injection time of 120 min. The collision energy was set to 30% and the underfill ratio to 0.6%. For dynamic exclusion, a timeout of 2.0 sec was chosen.

## Author Contributions

F.W., A.S., M.F.T., T.O.J., K.M. and O.P. conceived and planned the experiments. F.W., O.P., A.S., M.R. and B.S. performed experiments. A.K.N., W.E.T. and T.S.W. isolated hepatocytes. O.P., A.S., H.P., B.B., D.S. and F.W. performed data analysis. O.P. and K.M. prepared the manuscript. All authors discussed the results and commented on the paper.

## Supplementary Material

Supplementary Information Supplementary Info and Data

## Figures and Tables

**Table 1:** Intra- and inter-assay variances of the TXP-SRM assays targeting cytochrome P450 3A4, 3A5, 3A7, and multi-drug resistance protein 1

	Replicate [fmol μg^−1^]			Mean (n = 3)	Intra day variation [%]
	1	2	3		
CYP 3A4	13.0	15.1	15.3	14.5	9.0
CYP 3A5	0.7	0.8	0.8	0.7	7.6
CYP 3A7	0.3	0.3	0.3	0.3	10.2
MDR-1	1.1	1.2	1.2	1.2	6.9

	Replicate [fmol μg^−1^]			Mean (n = 3)	Inter day variation [%]
	1	2	3		
CYP 3A4	22.6	18.7	15.3	18.9	19.3
CYP 3A5	1.0	0.9	0.8	0.9	11.2
CYP 3A7	0.4	0.4	0.3	0.4	13.2
MDR-1	1.0	1.0	1.2	1.1	8.9
