# Supplementary material for: Indirect protein quantification of drug-transforming enzymes using peptide group-specific immunoaffinity enrichment and mass spectrometry
Source: Sci Rep. 2015 Mar 4;5:8759. doi: 10.1038/srep08759 (PMC4348618; doi:10.1038/srep08759)
Supplement: Supplementary Information — Supplementary Info and Data [file srep08759-s1.pdf]

Supplemental Material

Indirect protein quantification of drug-transforming enzymes using peptide group-specific immunoaffinity enrichment and mass spectrometry

Frederik Weiß[1], Anke Schnabel[2*], Hannes Planatscher[1], Bart H. J. van den Berg[1], Bettina Serschnitzki[2], Andreas K. Nuessler[3], Wolfgang E. Thasler[4], Thomas S. Weiss[5], Matthias Reuss[6], Dieter Stoll[7], Markus F. Templin[1], Thomas O. Joos[1], Katrin Marcus[2,#], Oliver Poetz[1#]

[1] NMI Natural and Medical Sciences Institute at the University of Tübingen, Markwiesenstr. 55, Reutlingen, Germany

[2] Department Functional Proteomics, Ruhr-Universität Bochum, Bochum, Germany

[3] Department of Traumatology, Eberhard Karls Universität Tübingen, Tuebingen, Germany

[4] Department of Surgery, Ludwig-Maximilians-University, Munich, Germany

[5] Department of Surgery, University of Regensburg, Regensburg, Germany

[6] Center Systems Biology, University of Stuttgart, Stuttgart, Germany

[7] University of Applied Sciences, Albstadt Sigmaringen, Germany

*present address: Protagen Protein Services GmbH, Otto-Hahn-Str. 15, 44227 Dortmund, Germany

#K.M. & O.P contributed equally

Corresponding author:

Dr. Oliver Poetz, NMI Natural and Medical Sciences Institute at the University of Tübingen, Markwiesenstr. 55, 72770 Reutlingen, Germany

E-mail: poetz@nmi.de

Phone: +49 7121 51530-802

Fax: +49 7121 51530-16

Supplemental Figure 1 Comparison of signals of endogenous cytochrome P450 (a) 3A5, (b) 3A7, and (c) MDR-1 peptide and synthetic standard. Ten µg hepatocyte protein extract was digested and spiked with 100 fmol synthetic standard. The samples were either analyzed by TXP-SRM or LC-SRM workflow. Ion chromatograms of the respective peptide fragments are shown.

Supplemental Figure 2 Comparison of signals of endogenous cytochrome P450 (a) 3A4/43, (b) 3A5, (c) 3A7, and (d) MDR-1 peptide and synthetic standard. One, five, ten or 25 µg hepatocyte protein extract was digested and spiked with 100 fmol synthetic standard. The samples were analyzed by TXP-SRM workflow. Integrated signals of the peptide fragments are plotted against the applied amount of protein extract.

Supplemental Figure 3. Binding capacity of the TXP antibody anti-LPNK. Synthetic non-labeled peptide standards of the cytochrome P450 (a) 3A5, (b) 3A7, and (c) MDR-1 sequences were mixed, each at 1 pmol per peptide (4 pmol peptide in total) and incubated with 1 µg antibody. The same amount of synthetic isotopically-labeled peptides was added to the elution buffer. Capacity was 1.6 pmol LPNK-peptide per µg antibody calculated by forming the signal ratio of labeled-to non-labeled peptide

Supplemental Figure 4 Linear range of **TXP immunoassays**. Peptide dilution series were prepared in low complex buffer. Endogenous 3A4/3A43- (a), 3A5- (b), 3A7- (c), and MDR1-peptides (d) were diluted by spanning concentration levels of four orders of magnitude while the concentration of isotopically-labeled peptides was kept constant. After immunoprecipitation, recoveries of the unlabeled peptides were determined. Data points within a confidence region of 100% ± 20% delineate the functional range of the TXP immunoassays.

Supplemental Table 1 Target proteins and corresponding signature peptides

Supplemental Table 2 Mass spectrometry parameters for target peptides

Supplemental Table 3 Integrated fragment ion signals of endogenous peptide and synthetic standard observed in 10 µg hepatocyte extract

Supplemental Table 4 Ratios of integrated fragment ion signals. One, five, ten or 25 µg hepatocyte protein extract was digested and spiked with 100 fmol synthetic standard. The samples were either analyzed by TXP-SRM or LC-SRM workflow. Integrated fragment ion signals for all synthetic as well as endogenous peptides were divided by each other.

Supplemental Table 5 Specificity of the anti-LPNK antibody (on- and off-target binding). We immunoprecipitated peptides from 50 µg digested hepatocyte protein extract and analyzed the peptide pool by nLC high-resolution mass spectrometry. Last four c-terminal amino acids are displayed in bold.

Supplemental Figure 1A

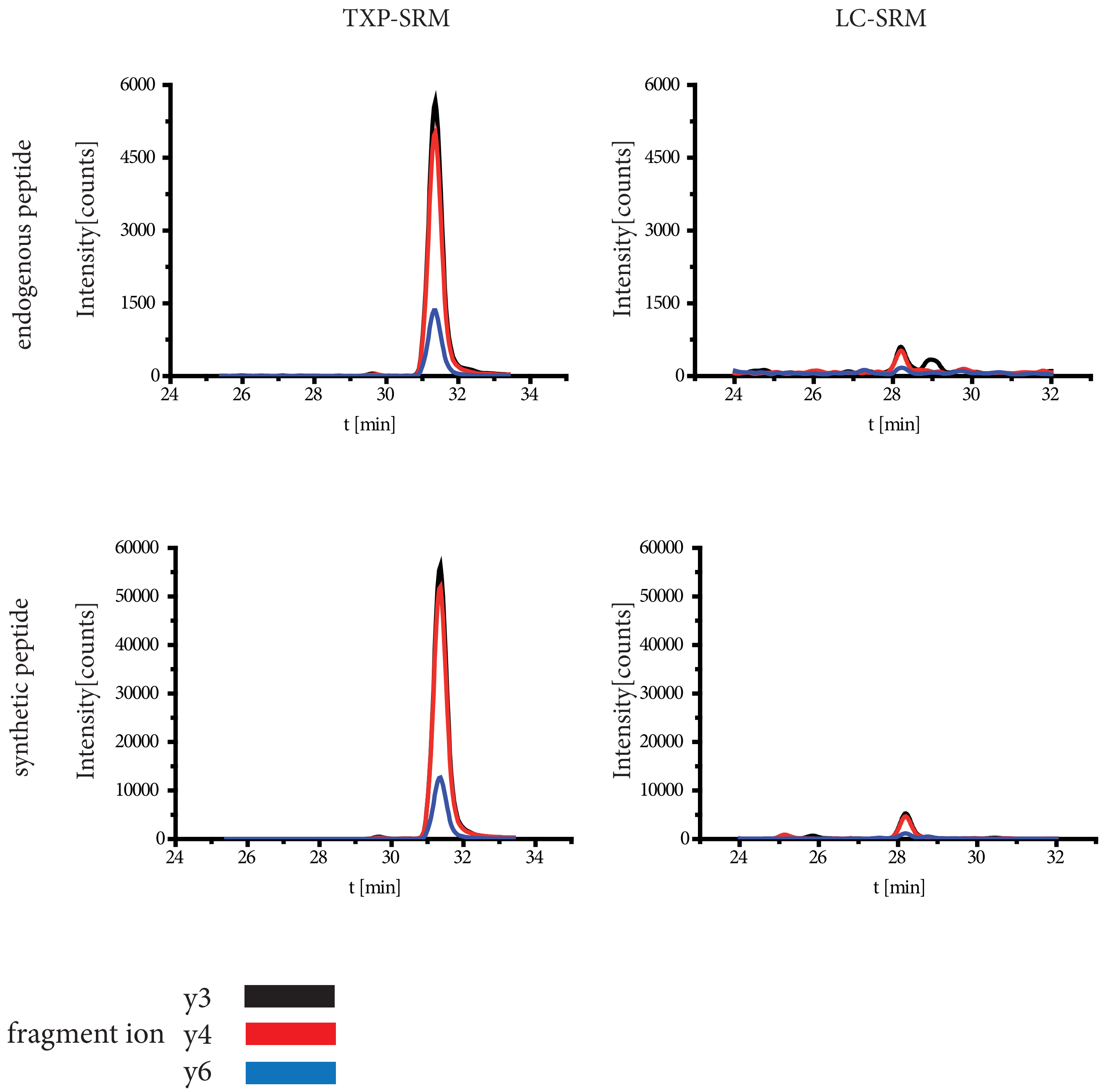

Supplemental Figure 1B

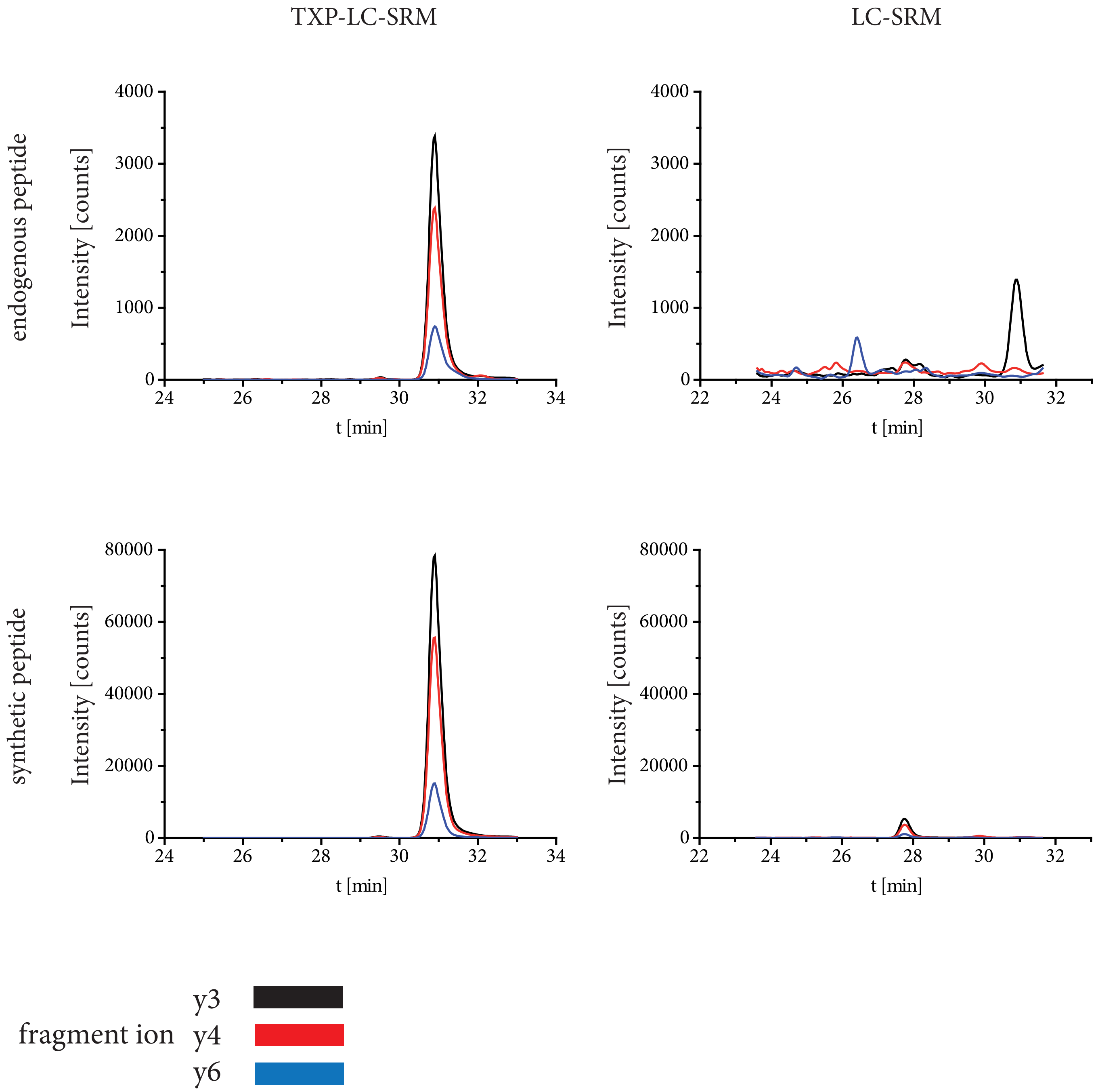

Supplemental Figure 1C

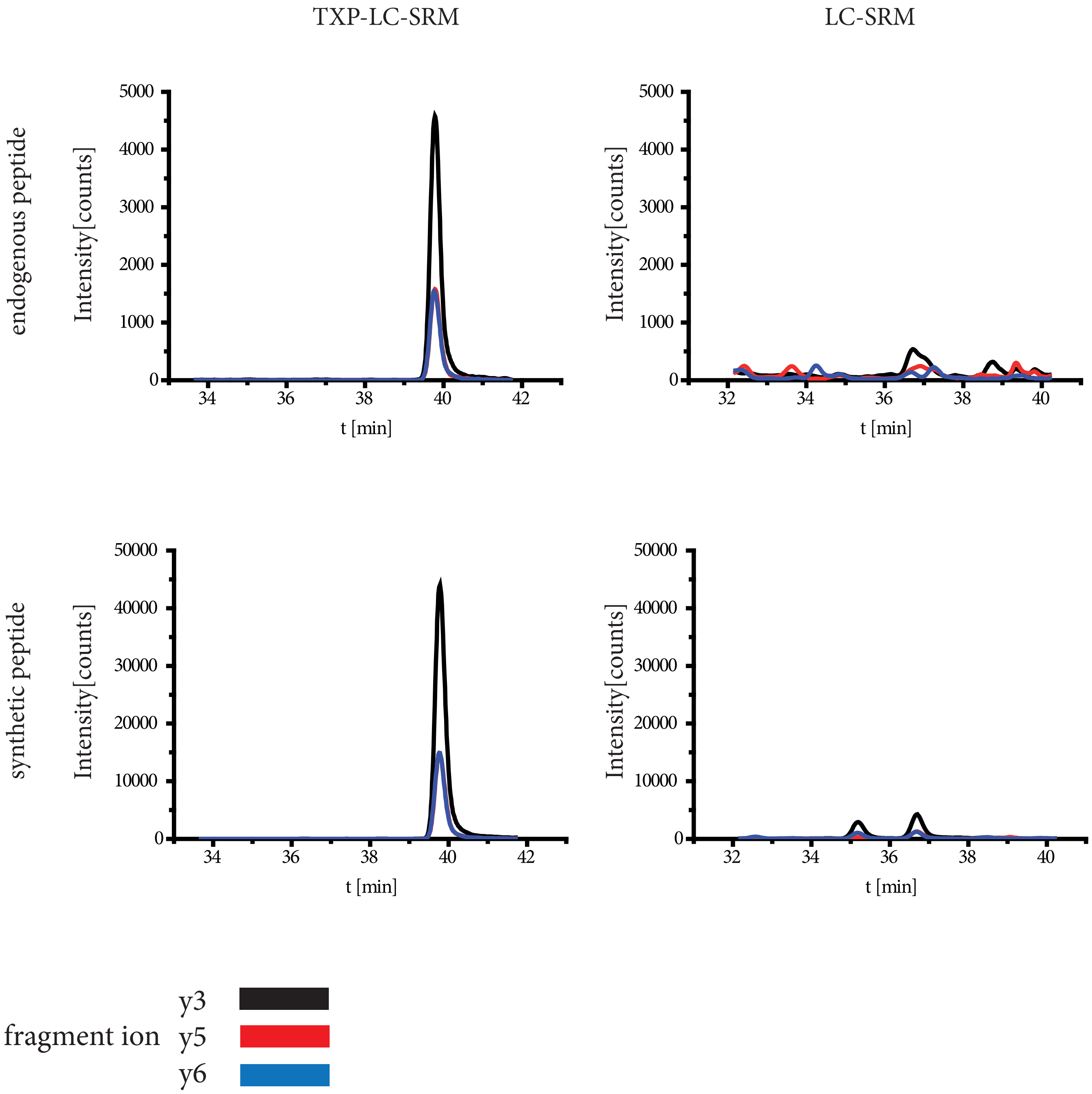

Supplemental Figure 2

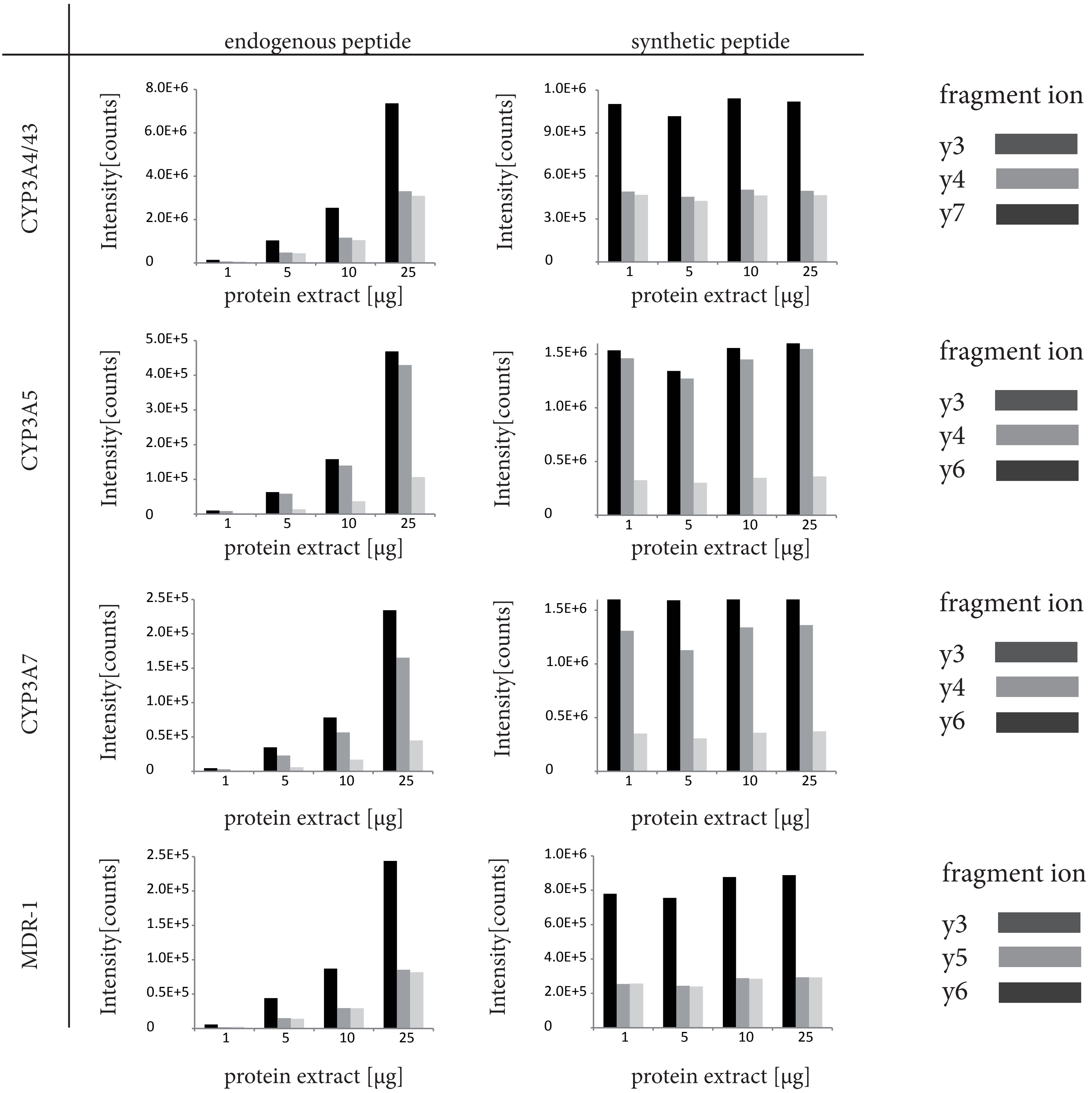

Supplemental Figure 3

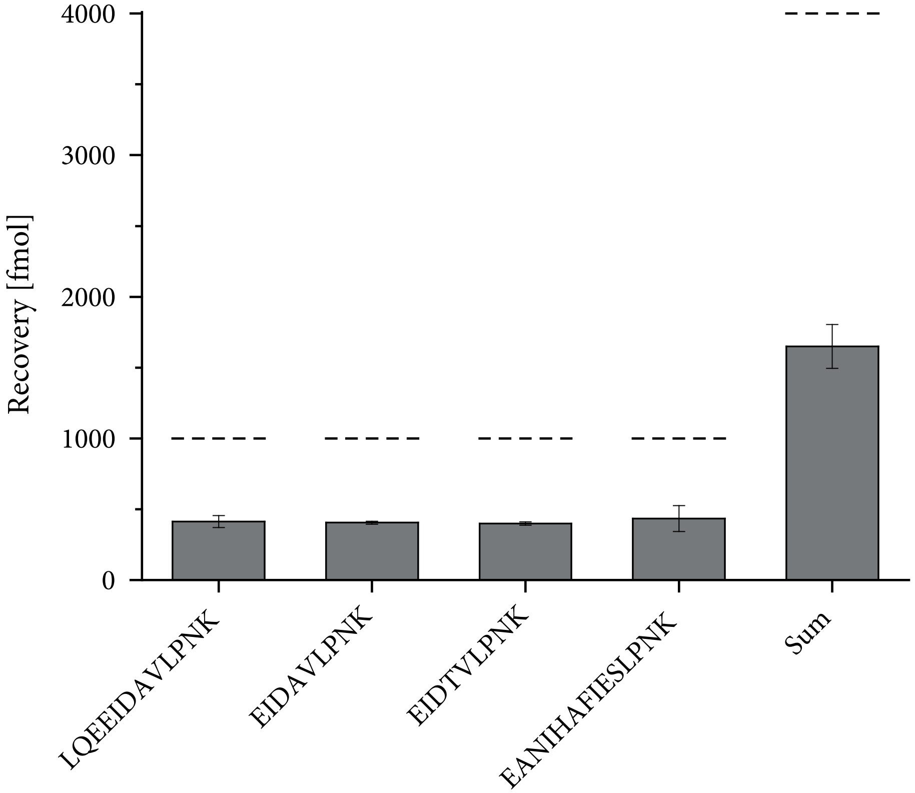

Supplemental Figure 4

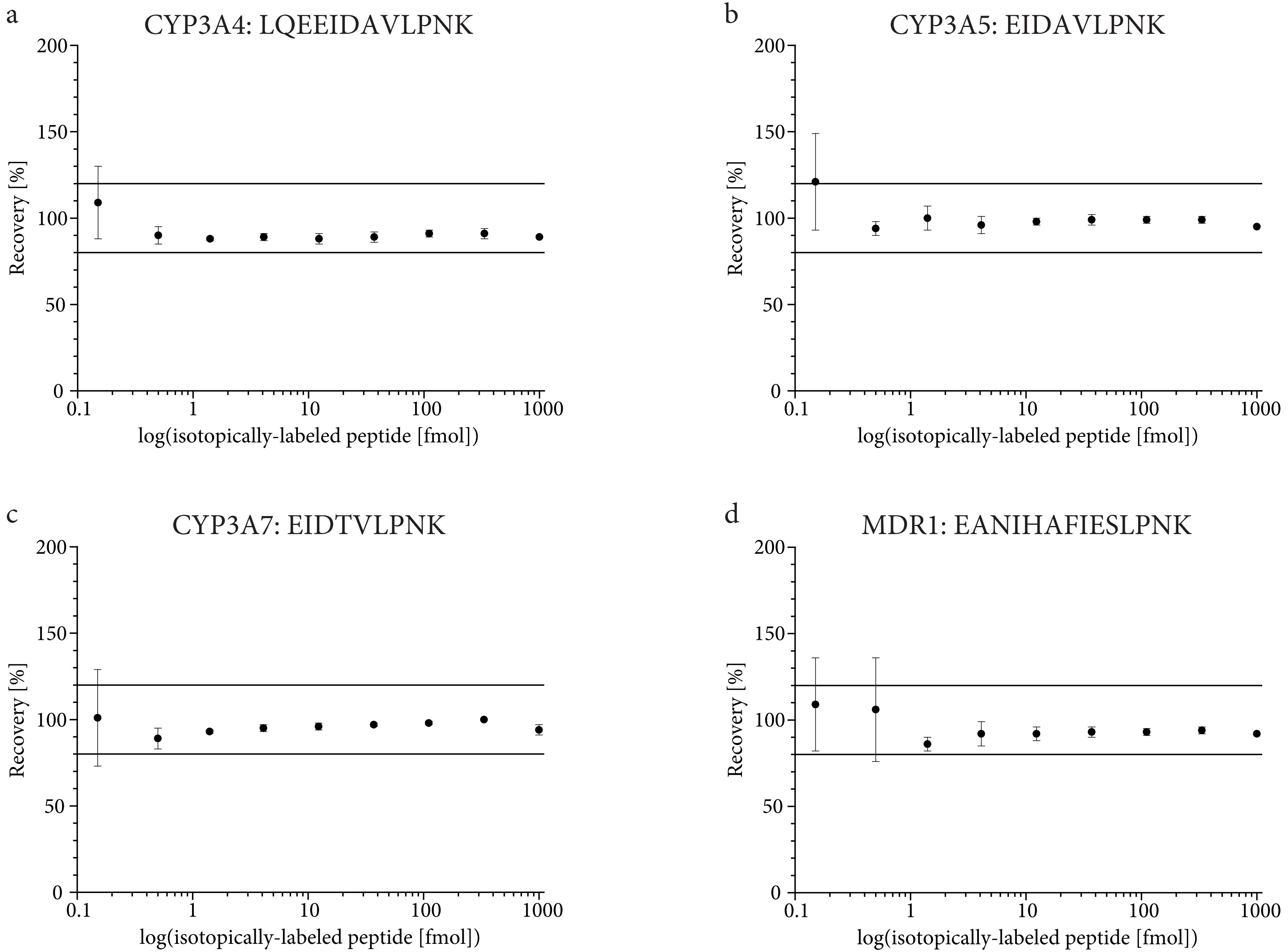

Supplemental Table 1 Target proteins and corresponding signature peptides

Protein name	Gene symbol	Signature peptide/ stable isotope standard	MH+ (mono)
Cytochrome P450 3A4/3A43*	CYP3A4	LQEEIDAVLPNK	1369,7
		LQEEIDAVLPN**<u>K</u>**	1377,7
Cytochrome P450 3A5	CYP3A5	EIDAVLPNK	999,6
		EIDAVLPN**<u>K</u>**	1007,6
Cytochrome P450 3A7	CYP3A7	EIDTVLPNK	1029,6
		EIDTVLPN**<u>K</u>**	1037,6
Multidrug resistance protein 1	ABCB1	EANIHAFIESLPNK	1584,9
		EANIHAFIESLPN**<u>K</u>**	1593,0

Bold and underlined amino acid symbols are stable isotopically labelled amino acid residues.

*Peptide sequence is also present in CYP3A43, but no or only very low expression in hepatocytes

Supplemental Table 2 SRM-MS parameters for target peptides

Protein name	Gene symbol	Accession Number	Peptid sequence	Parent ion m/z	Parent ion charge state	Fragment ion (m/z)	Ion type	Fragment Ion charge state	DP[#]	EP[‡]	CE[$]	CXP[§]
Cytochrome P450 3A4	CYP3A4	P08684	LQEEIDAVLPNK	684.9	2+	358.2	y3	1+	70	10	35	10
						471.3	y4	1+	70	10	35	13
						756.4	y7	1+	70	10	35	23
			LQEEIDAVLPN**K**	688.9	2+	366.2	y3	1+	70	10	35	10
						479.3	y4	1+	70	10	35	13
						764.4	y7	1+	70	10	35	23
Cytochrome P450 3A5	CYP3A5	P20815	EIDAVLPNK	499.8	2+	358.2	y3	1+	70	10	25	10
						471.3	y4	1+	70	10	25	13
						641.4	y6	1+	70	10	25	19
			EIDAVLPN**K**	503.8	2+	366.2	y3	1+	70	10	25	10
						479.3	y4	1+	70	10	25	13
						649.4	y6	1+	70	10	25	23
Cytochrome P450 3A7	CYP3A7	P24462	EIDTVLPNK	514.80	2+	358.2	y3	1+	70	10	25	10
						471.3	y4	1+	70	10	25	13
						671.4	y6	1+	70	10	25	20
			EIDTVLPN**K**	518.80	2+	366.2	y3	1+	70	10	25	10
						479.3	y4	1+	70	10	25	13
						679.4	y6	1+	70	10	25	21
Multidrug resistance protein 1	ABCB1	P08183	EANIHAFIESLPNK	528.30	3+	358.2	y3	1+	70	10	30.5	10
						558.3	y5	1+	70	10	30.5	17
						687.4	y6	1+	70	10	30.5	21
			EANIHAFIESLPN**K**	531.00	3+	366.2	y3	1+	70	10	30.5	10
						566.3	y5	1+	70	10	30.5	17
						695.4	y6	1+	70	10	30.5	21

Bold and underlined amino acid symbols are stable isotopically labelled amino acid residues.

DP, declustering potential
‡ EP, entrance potential
$ CE, collision energy
§ CXP, Collision Cell Exit Potential

Supplemental Table 3 Integrated fragment ion signals of endogenous peptide and synthetic standard observed in 10 μg hepatocyte extract

		TXP-SRM			LC-SRM		
Protein/peptide	transition	endogenous peptide [counts]	isotopic standard [counts]	calculated endogenous peptide [fmol]	endogenous peptide [counts]	isotopic standard [counts]	calculated endogenous peptide [fmol]
CYP3A4/	y3	2.5E+06	1.1E+06	223	3.0E+05	1.2E+05	251
LQEEIDAVLPNK	y4	1.2E+06	5.1E+05		1.2E+05	7.1E+04	
	y7	1.1E+06	4.6E+05		1.3E+05	3.3E+04	
CYP3A5/	y3	1.5E+05	1.6E+06	10	9.6E+03	1.2E+05	8
EIDAVLPNK	y4	1.4E+05	1.5E+06		8.0E+03	1.0E+05	
	y6	3.7E+04	3.5E+05		2.7E+03	1.9E+04	
CYP3A7/	y3	7.8E+04	1.9E+06	4	ND	1.2E+05	ND
EIDTVLPNK	y4	5.7E+04	1.3E+06		3.7E+03	7.5E+04	
	y6	1.7E+04	3.6E+05		ND	2.2E+04	
MDR1/	y3	8.7E+04	8.8E+05	10	1.6E+04	9.2E+04	ND
EANIHAFIESLPNK	y5	3.0E+04	2.9E+05		ND	2.8E+04	
	y6	3.0E+04	2.9E+05		2.6E+03	2.7E+04	

Supplemental Table 4 Fragment ion signal observed in 1, 5, 10 and 25 µg hepatocyte extract

			TXP-SRM							LC-SRM						
	Protein	Ratio	1 µg	5 µg	10 µg	25 µg	MW	stdv	cv [%]	1 µg	5 µg	10 µg	25 µg	MW	stdv	cv [%]
Endogenous peptide	CYP3A4	y3/y4	2.1	2.2	2.2	2.2	2.2	0.1	2.9	2.3	2.6	2.5	2.5	2.5	0.1	4.6
		y3/y7	2.4	2.3	2.4	2.4	2.4	0.0	1.2	2.3	2.5	2.3	2.2	2.3	0.1	5.7
		y4/y7	1.1	1.1	1.1	1.1	1.1	0.0	2.9	1.0	1.0	0.9	0.9	0.9	0.0	5.3
	CYP3A5	y3/y4	1.2	1.1	1.1	1.1	1.1	0.0	3.8	0.9	1.0	1.2	1.1	1.1	0.1	12.2
		y3/y6	4.4	4.5	4.3	4.4	4.4	0.1	2.3	n.d.	3.7	3.6	2.4	3.2	0.7	22.3
		y4/y6	3.8	4.2	3.8	4.0	3.9	0.2	5.3	n.d.	3.9	3.0	2.2	3.0	0.8	28.4
	CYP3A7	y3/y4	1.5	1.5	1.4	1.4	1.5	0.1	4.3	n.d.	n.d.	n.d.	n.d.	n.d.	n.d.	n.d.
		y3/y6	4.2	5.8	4.6	5.2	5.0	0.7	14.4	n.d.	n.d.	n.d.	n.d.	n.d.	n.d.	n.d.
		y4/y6	2.8	3.8	3.3	3.7	3.4	0.4	13.1	n.d.	n.d.	n.d.	n.d.	n.d.	n.d.	n.d.
	MDR1	y3/y5	3.0	2.9	2.9	2.9	2.9	0.1	2.3	n.d.	n.d.	n.d.	n.d.	n.d.	n.d.	n.d.
		y3/Y6	2.3	3.1	2.9	3.0	2.8	0.4	13.3	2.1	8.0	6.2	n.d.	5.4	3.0	55.8
		y5/y6	0.8	1.1	1.0	1.0	1.0	0.1	14.8	n.d.	n.d.	n.d.	n.d.	n.d.	n.d.	n.d.
	Protein	Ratio	1 µg	5 µg	10 µg	25 µg	MW	stdv	cv [%]	1 µg	5 µg	10 µg	25 µg	MW	stdv	cv [%]
Synthetic peptide	CYP3A4	y3/y4	2.2	2.2	2.3	2.3	2.2	0.0	0.5	2.3	1.8	1.7	2.5	2.1	0.4	18.9
		y3/y7	2.4	2.4	2.5	2.4	2.4	0.0	1.8	2.3	3.3	3.6	3.4	3.1	0.6	18.6
		y4/y7	1.0	1.1	1.1	1.1	1.1	0.0	1.5	1.0	1.8	2.1	1.3	1.6	0.5	32.1
	CYP3A5	y3/y4	1.0	1.1	1.1	1.1	1.1	0.0	0.9	1.1	1.1	1.1	1.1	1.1	0.0	1.7
		y3/y6	4.7	4.4	4.5	4.5	4.5	0.1	2.7	4.5	5.8	6.2	4.8	5.4	0.8	15.1
		y4/y6	4.5	4.2	4.2	4.3	4.3	0.1	3.4	4.1	5.3	5.6	4.2	4.8	0.7	15.5
	CYP3A7	y3/y4	1.4	1.4	1.3	1.4	1.4	0.0	1.7	1.5	1.6	1.6	1.6	1.6	0.1	4.5
		y3/y6	5.1	5.2	5.2	5.1	5.2	0.0	0.8	5.5	5.8	5.3	5.7	5.6	0.2	3.8
		y4/y6	3.8	3.7	3.9	3.7	3.8	0.1	2.3	3.7	3.7	3.4	3.5	3.6	0.2	5.0
	MDR1	y3/y5	3.1	3.1	3.0	3.0	3.1	0.0	0.9	3.1	3.3	3.3	3.5	3.3	0.2	4.6
		y3/Y6	3.0	3.1	3.1	3.0	3.1	0.1	1.8	3.2	3.3	3.5	2.8	3.2	0.3	9.4
		y5/y6	1.0	1.0	1.0	1.0	1.0	0.0	1.3	1.0	1.0	1.1	0.8	1.0	0.1	12.2

*n.d. not determined due to missing values

Supplemental Table 5

#	Sequence	C-terminus	Replicate 1			Replicate 2			Replicate 3		
			PSM	Mascot Score	Sequest	PSM	Mascot Score	Sequest	PSM	Mascot Score	Sequest
1	AAILPTSIFLTNK	LTNK	0			1	49.23	2.16	1	18.17	
2	AALEALGScLNNK*	LNNK	1	21.14		1	25.97		1	25.43	1.77
3	AAYIYFTDFcPDNLFNK	LFNK	1	32.87		1	61.24	2.18	1	46.77	
4	AISNSHYVLELTLPNIYVTLPNK	LPNK	1	36.73	1.94	1	17.26		0		
5	APVLFNK	LFNK	1	16.51		0			1	13.01	
6	AVDcFTAALSVRPNDYLLWNK	LWNK	1	16.29		2	14.91	2.07	1	19.57	
7	AVLEALGScLNNK	LNNK	1	18.56	2.25	1	37.9		1	84.09	3.01
8	AVMVSNILLINK	LINK	1	51.78	1.89	2	32.51		1	34.96	1.71
9	AWITNAWEASAAVVFASTDRALQNK	LQNK	2	101.74	3.70	2	64.14	4.89	4	96.6	5.74
10	cIYcGFcQEAcPVDAIVEGPNFEFSTETHEELLYNK	LYNK	1	45.2	4.47	1	40.32	5.28	0		
11	cLmANSTWGYPFHDGDYLNDEALWNK#	LWNK	1	34.07	2.75	1	39.46	3.28	3	48.09	2.51
12	cTLTSIPQTQALLNK	LLNK	1	17.42	1.63	0			0		
13	cTSNPAEIAMFLEPLDLPNK	LPNK	1	60.09	2.54	0			1	37.2	2.29
14	DAIILIFANK	FANK	0			0			1	17.31	
15	DATNVGDEGGFAPNILENK	LENK	4	88.86	4.78	2	66.79	3.28	3	102.52	5.21
16	DAVLLVFANK	FANK	1	14.91		0			1	33.35	
17	DGIILcELINK	LINK	1	49	2.10	1	49.79	1.98	1	46.47	2.37
18	DNHLVHLPLTLILVNK	LVNK	1	35.67		1	33.89	2.33	1	44.92	1.79
19	DPENFPFVVLGNK	LGNK	2	57.64	3.45	3	76.13	3.41	3	53.59	3.98
20	DYFAEQVYWSPLLNK	LLNK	1	58.42	2.08	1	43.92	1.84	1	44.77	2.00
21	EAISDAALEATENEPLNKETEEMcVMLVSNLPNK	LPNK	0			1	13.12		1	25.69	
22	EANIHAFIESLPNK	LPNK	2	73.58	4.53	2	71.84	3.88	2	77	4.41
23	EELDSLMTDETIANVPILILGNK	LGNK	1	46.26		3	80.57	3.01	3	116.96	4.09
24	EIIEHGIELFNK	LFNK	1	25.68		0			0		
25	ELHINLIPNK	IPNK	5	38.57	3.23	5	32.03	3.09	5	33.38	3.21
26	ETEEMcVMLVSNLPNK	LPNK	1	40.56		0			1	31.38	
27	EVQMHSFPLQFTLHcALLANK	LANK	1	30.95		2	42.29	2.19	1	48.74	3.36
28	EVYQGSEVEcTVSSLLPGK	LPGK	0	43.22	2.36	1	16.75		0		

#	Sequence	C-terminus	Replicate 1			Replicate 2			Replicate 3		
			PSM	Mascot Score	Sequest	PSM	Mascot Score	Sequest	PSM	Mascot Score	Sequest
29	FAHTNVESLVNEYDDNGEGIILFRPSHLTNK	LTNK	3			3	64.41	5.92	3	45.46	6.36
30	FAVFGLGNK	LGNK	1	42.72	1.88	0			1	17.01	1.56
31	FAWVEADGVNcIMGVLSNK	LSNK	0			1	17.85		0		
32	FMQTFVLAPEGSVPNK	VPNK	0			0			1	17.44	
33	FNVLHWHIVDDQSFPYQSITFPELSNK	LSNK	3	56.53	4.33	2	67.83	5.21	3	75.01	5.73
34	FRSETITEEELVGLMNK	LMNK	2	36.17		1	52.89	3.15	3	35.81	
35	GcQLTITFSVPNK	VPNK	2	90.58	3.42	2	90.71	3.26	2	85.23	4.00
36	GLTVQQWDALYTDLPNR	LPNR	1	41.74		0			1	80.34	2.94
37	GMEELIPLVNK	LVNK	1	29.29	1.59	0			1	22.04	
38	GTSIVVPEPLHFLLPGK	LPGK	1	14.73		1	23.11		0		
39	GTVIIIANHGDRIDIPPGAVLENK	LENK	3	22.76		8	31.8	3.02	7	32.34	3.98
40	HQAQIDHYLGLANK	LANK	1	73.4	4.14	1	66.11	2.26	1	78.4	4.38
41	HTEVPTGTcPVDPFEAQWAALENK	LENK	0			0			1	40.64	1.55
42	HVDLLINK	LINK	1		1.89	0			0		
43	IDIPPGAVLENK	LENK	8	43.99	2.46	7	34.11		6	36.45	1.84
44	IEDLGAAMEEALILDNK	LDNK	0			1	18.66	1.37	0		
45	ILPTLEAVAALGNK	LGNK	0			0			1	66.56	2.46
46	INEIVYFLPFcHSELIQLVNK	LVNK	1	19.22		2	26.83	2.27	2	40.73	2.16
47	IQDLPPVDLSLVNK	LVNK	0			0			1	17.56	
48	KIEDLGAAMEEALILDNK	LDNK	2	30.81		2	59.1	1.88	2	52.38	1.81
49	KPPTFGDASVIALELLNSGYEFDEGSIIFNK	IFNK	1	31.17		1	53.86	2.38	2	63.98	4.40
50	LDFLRPYTVPNK	VPNK	3	23.96	3.27	3	19.7	2.80	1	29.24	3.34
51	LKQDPYLVNFFLENK	LENK	0			1	36.62	1.94	1	22.8	
52	LLELLWNK	LWNK	2	31.38	1.91	1	20.43		1	21.87	
53	LLLFLSTELQASQILQNK	LQNK	1	21.15		1	29.91		1	43.31	1.55
54	LNLAFVANLFNK	LFNK	1	55.08		2	79.42	3.87	2	79.14	3.37
55	LQEEIDAVLPNK	LPNK	3	78.27	4.02	2	74.79	3.64	2	81.15	3.87
56	LQFDYGVYLLNK	LLNK	0			0			1	19.96	
57	LTLPNGEPVPcLLLANK	LANK	1	31.25	1.97	1	48.11	2.42	2	39.12	2.29

#	Sequence	C-terminus	Replicate 1			Replicate 2			Replicate 3		
			PSM	Mascot Score	Sequest	PSM	Mascot Score	Sequest	PSM	Mascot Score	Sequest
58	LTVPDDIAANcIYLNIPNK	IPNK	15	95.57	5.09	14	101.24	5.36	14	89.33	5.33
59	LVNLQHLDLLNNK	LNNK	1	16.51		0			1	27.74	
60	LYQYPNFAGPHAALANK	LANK	2	32.71	2.45	0			0		
61	MASIYSEEGNIEHAFILYNK	LYNK	2	33.25	1.88	2	63.26	2.43	0		
62	MLSLAEQQLVDcAQDFNNHGcQGGLPSQAFEYILYNK	LYNK	5	41.7	5.89	4	46.68	7.66	4	42.97	8.09
63	MVLSVFSPYWLINK	LINK	0			0			1	13.66	
64	NELHNLLDKPQLQGIPVLVLGNK	LGNK	1	63.56	2.08	3	48.97	2.66	3	71.37	4.28
65	NFHVFYQLLSGASEELLNK	LLNK	3	67.03	3.59	2	80.97	4.78	5	49.78	3.93
66	NLSLSGHVGFDSLPDQLVNK	LVNK	0			1	25		0		
67	NSSSGVSPLYSPVSFcGIPVGVLQNK	LQNK	1	32.26		1	16.96		0		
68	QAGLYSISLPNK	LPNK	1	46.86	2.63	0			0		
69	QILLGPNTGLSGGMPGALPSLPGK	LPGK	0			0			1	19.47	
70	QSDVMIVAGTLTNK	LTNK	1	57.63	1.95	0			1	28.17	
71	RAVMVSNILLINK	LINK	1	31.65	1.57	1	35.64		2	34.02	2.00
72	SEEELLVIFNK	IFNK	1	40	1.52	1	40.65		1	37.36	1.60
73	SETITEEELVGLMNK	LMNK	5	121.33	4.59	6	121.35	4.74	9	121.34	4.57
74	SGFWTIWLWNK	LWNK	0			0			1	31.69	
75	SIQETLGESGSLLPNK	LPNK	2	77.16	3.32	0			1	91.45	3.77
76	SLDWQIDVDLLNK	LLNK	0			1	66.54	1.96	1	24.35	2.21
77	SLIEVYMRPLSNK	LSNK	1	20.78		0			0		
78	SPPILPPHLLQVILNK	ILNK	0			0			1	17.68	
79	SQEFYELLNK	LLNK	0			0			1	21.37	
80	TAGWNIPMGLLYNK	LYNK	15	56.44	4.50	19	53.71	4.55	21	63.76	4.53
81	TLAFTSVDLTNK	LTNK	0			0			1	14.48	
82	TLVLLMGKEGVHGGLINK	LINK	0			0			1	21.08	
83	TQHGVLSQQFVELINK	LINK	1	16.64		0			0		
84	TSGTLISFIYPAQNPELLNK	LLNK	7	87.47	5.78	9	78.37	6.07	10	70.46	5.93
85	TVGEIMWGYKDPLVNLINK	LINK	1	20.92		0			1	20.72	

#	Sequence	C-terminus	Replicate 1			Replicate 2			Replicate 3		
			PSM	Mascot Score	Sequest	PSM	Mascot Score	Sequest	PSM	Mascot Score	Sequest
86	VAcITEQVLTLVNK	LVNK	3	83.5	3.87	3	71.11	3.57	6	63.62	3.90
87	VASPGSTIDTAPLSSVDSLINK	LINK	0			0			1	16.14	
88	VDGLLTccSVLINK	LINK	1	61.19	3.61	3	55.93	2.98	3	53.94	3.39
89	VELNALMTDETISNVPILILGNK	LGNK	1	57.78	2.00	2	104.02	4.01	2	86.3	3.44
90	VHLPNGSPIPAVLLANK	LANK	2	37.82	1.84	3	30.75	1.77	2	26.22	1.65
91	VILNAATLTGAmDVALGSGATGVFTNSSWLWNK	LWNK	6	116.57	6.37	5	102.88	5.43	4	109.58	4.95
92	VKYEETVFYGLQYILNK	ILNK	0			1	39.97		0		
93	VNAMTFTFDNVLPGK	LPGK	2	43.47	1.53	1	41		1	50.51	2.58
94	VNVNLLIYLLNK	LLNK	2	79.31	3.40	2	78.94	3.57	2	73.94	3.31
95	VSGQGRDQALNLLNK	LLNK	0			0			2	15.88	
96	VVTSEALcGVPVLVLANK	LANK	2	92.5	4.21	3	50.16	2.80	3	63.28	3.24
97	WYLFGMGNEVDVHAAFFHGQALTNK	LTNK	0			1	17.59		2	25.64	1.91
98	YAQVKPDGTYVKPLSNK	LSNK	3	37.01	1.94	1	15.78		1	16.08	
99	YDGIILPGK	LPGK	1	27.42		1	13.55		1	16.58	
100	YDIDLPNK	LPNK	1		1.55	0			0		
101	YDKLTVPDDIAANcIYLNIPNK	IPNK	3	69.91	5.07	3	73.08	5.73	5	73.59	5.45
102	YEDFVVDGFNVLYNK	LYNK	2	61.53	3.89	3	47.38	2.85	3	50.36	3.31
103	YFDLGLPNR	LPNR	3	41.16	2.28	2	34.07	2.22	4	43.35	1.92
104	YFTFPFEEIDFSMEESMIHLPNK	LPNK	15	68.23	6.78	15	70.76	7.18	17	73.75	6.33
105	YPDFAAGWALSIPLVNK	LVNK	2	19.46		1	22.07		1	14.12	

*c carbamidomethylated cysteine # m oxidized and non-oxidized methionine
